# Insulin Enhances Migration and Invasion in Prostate Cancer Cells by Up-Regulation of FOXC2

**DOI:** 10.3389/fendo.2019.00481

**Published:** 2019-07-17

**Authors:** Phoebe L. Sarkar, Wendy Lee, Elizabeth D. Williams, Amy A. Lubik, Nataly Stylianou, Ali Shokoohmand, Melanie L. Lehman, Brett G. Hollier, Jennifer H. Gunter, Colleen C. Nelson

**Affiliations:** ^1^Queensland University of Technology (QUT), Australian Prostate Cancer Research Centre-Queensland, Institute of Health and Biomedical Innovation, School of Biomedical Sciences, Faculty of Health, Translational Research Institute, Brisbane, QLD, Australia; ^2^Vancouver Prostate Centre, Department of Urologic Sciences, University of British Columbia, Vancouver, BC, Canada

**Keywords:** hyperinsulinemia, prostate cancer, androgen deprivation, invasion, epithelial to mesenchymal transition (EMT), FOXC2

## Abstract

Androgen deprivation therapy (ADT) is the standard treatment for advanced prostate cancer (PCa), yet many patients relapse with lethal metastatic disease. With this loss of androgens, increased cell plasticity has been observed as an adaptive response to ADT. This includes gain of invasive and migratory capabilities, which may contribute to PCa metastasis. Hyperinsulinemia, which develops as a side-effect of ADT, has been associated with increased tumor aggressiveness and faster treatment failure. We investigated the direct effects of insulin in PCa cells that may contribute to this progression. We measured cell migration and invasion induced by insulin using wound healing and transwell assays in a range of PCa cell lines of variable androgen dependency (LNCaP, 22RV1, DuCaP, and DU145 cell lines). To determine the molecular events driving insulin-induced invasion we used transcriptomics, quantitative real time-PCR, and immunoblotting in three PCa cell lines. Insulin increased invasiveness of PCa cells, upregulating Forkhead Box Protein C2 (FOXC2), and activating key PCa cell plasticity mechanisms including gene changes consistent with epithelial-to-mesenchymal transition (EMT) and a neuroendocrine phenotype. Additionally, analysis of publicly available clinical PCa tumor data showed metastatic prostate tumors demonstrate a positive correlation between insulin receptor expression and the EMT transcription factor FOXC2. The insulin receptor is not suitable to target clinically however, our data shows that actions of insulin in PCa cells may be suppressed by inhibiting downstream signaling molecules, PI3K and ERK1/2. This study identifies for the first time, a mechanism for insulin-driven cancer cell motility and supports the concept that targeting insulin signaling at the level of the PCa tumor may extend the therapeutic efficacy of ADT.

## Introduction

In advanced prostate cancer (PCa), the dependency of tumor cells on androgen receptor (AR) signaling has made targeting this pathway a primary focus of drug development, with patients receiving androgen deprivation therapy (ADT) as first line treatment for recurrent disease. However, many patients relapse with rising serum levels of prostate specific biomarker PSA and lethal metastases. Rising PSA triggers commencement of AR targeted therapies (e.g., enzalutamide), but these confer only modest gains in overall survival of 4–5 months (1). Relapsing tumors are also estimated to consist of 10–30% neuroendocrine (NE) tumors, the deadliest subset of the disease (2, 3).

Previous research has focused on mechanisms by which androgens drive PCa growth, survival and metastasis (4–6). These results have rationalized the development of therapies which continue to target the androgen axis, such as enzalutamide, and subsequently improved patient survival. However, continued targeting extends the duration of AR suppression in patients and emerging data suggests prolonged AR suppression may have inadvertent consequences on progression. Studies have shown an emergence of metastatic and NE tumors post-ADT and enzalutamide therapy that may be attributed to activation of key cell plasticity mechanisms that increase cell invasion and migration (7). Androgens confer an epithelial differentiation signal in prostate cells, which is lost upon AR suppression. This leads to cellular de-differentiation and trans-differentiation, resulting in the expression of numerous epithelial-to-mesenchymal transition (EMT) (7–9), NE (10–12), and stemness (7) markers. Interplay between these states is the subject of active research. Nevertheless, to consider AR suppression alone as the major effector of PCa plasticity presents an incomplete clinical picture as ADT leads to concurrent systemic hormonal and metabolic changes in patients that are associated with poor outcome.

The rapid appearance of features of the metabolic syndrome, including the development of insulin resistance and hyperinsulinemia, are well-documented side-effects of ADT (13), and are exacerbated with addition of second line treatments (14). Hyperinsulinemia develops in PCa patients within 2 weeks of commencing ADT (15) and is a direct consequence of decreased serum testosterone levels (16). Hyperinsulinaemia has also been associated with increased PCa specific mortality (17–20) and more rapid treatment failure (18). Immunohistochemical analysis of PCa tumors has shown that insulin receptor expression increases with Gleason grade (21, 22) and duration of ADT (23), and we have previously reported insulin to signal directly to PCa cells to increase *de novo* steroidogenesis (24).

Insulin signaling, however, has a myriad of functional responses in cells depending on context and timing (16). In cancer cells, serum from obese mice and humans, which have a number of altered metabolites including high levels of insulin, has been shown to increase cell migration in melanoma and PCa cells (25, 26). As androgen deprivation and AR inhibition can activate cell motility and plasticity mechanisms in PCa, we hypothesized that insulin may be accelerating these processes during androgen deprivation. The objective of this study was to examine the effect of insulin on cell plasticity in a model of androgen deprived PCa cells. We identified that insulin drives the adoption of EMT and NE features in PCa cells by upregulation of transcription factor Forkhead Box Protein C2 (FOXC2), and that this phenotype change coincides with increased migration and invasion by the cells. Increased invasion is blocked by targeting the insulin receptor (IR) and does not occur in the presence of androgen. Inhibition of FOXC2 phenocopies these insulin effects. Transcriptomic databases from clinical samples reveal FOXC2 and IR expression are positively correlated in primary and metastatic human PCa tissue, but not in benign prostate tissue, suggesting a relationship between insulin and FOXC2 in the development and progression of PCa. Thus, this study reports for the first time the mechanism by which insulin may increase the invasive potential of tumor cells. These novel results support the case for controlling ADT-induced hyperinsulinemia in PCa, the targeting of which is currently under investigation in a number of clinical trials [NCT02614859, NCT01796028, NCT01677897, (27)]. Our results also indicate that inhibitors to PI3K and MEK1/2 downstream of IR may be useful in suppressing insulin induced adaptive plasticity in PCa.

## Methods

### Cell Lines and Culture

LNCaP (passage 30–45), 22RV1 (passage 20–30), and DU145 (passage 5–15) were obtained from American Type Culture Collection (ATCC, Manassas, VA, USA). The cells were authenticated by STR analysis and were tested regularly for mycoplasma by PCR. Cells were maintained in phenol red-free RPMI-1640 medium containing L-Glutamine (Life Technologies, Carlsbad, USA) and 10% fetal bovine serum (FBS; Invitrogen). DuCaP cells (passage 8–15) were provided by Matthias Nees from the VTT Technical Research Center of Turku, Finland, and were maintained in phenol red-free Gibco RPMI-1640 medium containing L-Glutamine with 10% FBS. HEK293T cells (ATCC) and Chinese Hamster Ovary cells over-expressing Insulin Receptor, CHO.IR cells (passage 10–15) (kind gift of Prof Jon Whitehead, University of Lincoln, UK), were maintained in DMEM with L-Glutamine and 2.438 g/L sodium bicarbonate (Life Technologies) and 10% FBS. All cells were grown at 37°C in a humidified atmosphere of 5% CO_2_. LNCaP, DuCaP, and 22RV1 are AR-positive, cell lines. DuCaP cells contain high levels of wild-type AR and are androgen dependent. LNCaP cells have a mutation in their AR (T877A) which renders the receptor promiscuous to progesterone, estrogen and the antiandrogen bicalutamide (28). 22RV1 express a number of known AR variants associated with androgen independence (29). DU145 cells are AR negative and do not respond to androgens.

### Treatment With Hormones and Inhibitors

Cells were seeded in RPMI (10% FBS) and incubated overnight. Media was changed to RMPI containing 10% charcoal-stripped serum (CSS, Sigma Aldrich, Castle Hill, NSW, Australia) and incubated for 48 h to simulate androgen deprivation. Following this, cells were transferred to serum-free media with 0.2% BSA (Sigma-Aldrich, St. Louis, MO, USA) and treated with vehicle (ethanol; EtOH) or the following inhibitors as described: insulin (10 nM), dihydrotestosterone (DHT; 10 nM), AR antagonists, bicalutamide (10 μM) or enzalutamide (10 μM), IR/IGF-1R small peptide inhibitor BMS 745807 (500 nM), insulin-like growth factor 1 receptor (IGF-1R) antibody antagonist CP751871 (5 μg/mL), MAPK kinase inhibitors, PD98059 (10 μM), or U0126 (10 μM), PI3K inhibitor LY294002 (5 μM), and cell cycle inhibitor mitomycin C (MMC; 10 μg/mL).

### Generation of shRNA Stable Cell Lines

pTRIPZ lentiviral doxycycline-inducible shRNA containing one of three microRNA-adapted shRNA against INSR (cloneID:sequence V3THS_319895:5′-GGAAAGAATCAAGGAGG-3′, V3THS_319891:5′-GGAAAGAATCAAGGAGG-3′, VSTHS_319890:5′-GGAAAGAATCAAGGAGG-3′), and ZEB1 (cloneID:sequence V2THS_116663: 5′-GGAAAGAATCAAGGAGG-3′, V2THS_226625:5′-GGAAAGAATCAAGGAGG-3′, V2THS_116659:5′-GGAAAGAATCAAGGAGG-3′) were purchased (Dharmacon, GE, Millenium Sciences, Melbourne, Australia). FOXC2 shRNA have been previously described (30). Virus was produced by cotransfecting pTRIPZ and MISSION RNAi Lentiviral Packaging Mix (Sigma-Aldrich), according to the manufacturer's instructions. Briefly, HEK293T cells were transfected with 185 μl of serum-free DMEM containing 2 μl of FUGENE, 1.8 μg of pCMV gag/pol (pUMVC), 0.2 μg of pCMV-VSVG, and 2.0 μg of pTRIPZ construct. After 24 h the 293T cells were switched to 7 mL fresh serum free DMEM and viral particles allowed to accumulate in the media for a further 48 h before collected and filter sterilized. Lentivirus-containing media was added directly to LNCaP cells seeded in 6-well plates at 9 ×10^4^ cells/well. After 24 h, cells were changed into RPMI (5% FBS) and supplemented with 2 μg/mL puromycin (Invitrogen) (selection) or 1 μg/mL puromycin (maintenance). These constructs were created in LNCaP (passage 22–25) and used within five passages. Optimal knockdown of gene expression was confirmed using qRT-PCR and Western blot. shFOXC2 cells were prepared in LNCaP cells using shRNA-expressing pLKO lentivirus system (OpenBiosystems) as previously described (30). FOXC2 shRNA targeting sequences were CCTGAGCGAGCAGAATTACTA (pLKO5) and GCGGGAGATGTTCAACTCCCA (pLKO4). The shRNA sequences targeting firefly luciferase (shCntrl) in the pLKO vector were used as controls. The stable suppression of target genes was achieved by selection of cells in 2 μg/ml puromycin.

### Wound-Healing Migration Assay

Cells were grown to confluence in 96-well Imagelock plates (Essence Biosciences, Ann Arbor, MI, USA) then changed to CSS for 48 h. Cells were pretreated with MMC to block proliferation 2 h prior to addition of hormones and inhibitors. Wounds were created using the Incucyte Wound Maker (Essence Biosciences) according to manufacturer's instructions and treatments added. Wounds were imaged using Incucyte every 2 h at ×20 magnification (Essence Biosciences). Experiments contained 6–12 replicates in three independent experiments for each treatment in each cell line.

### Transwell Migration Assay

Transwell migration assays were performed in 24-well plates using MilliCell culture inserts with 8 μm pore size polycarbonate filters of 12 mm diameter (Merck Millipore, Billerica, USA). Cells were pre-treated with hormones and inhibitors for 24 h in serum-free RPMI, before being split and seeded, maintaining treatments in triplicate at 2.5 × 10^5^ cells per insert with RPMI containing 10% FBS as the chemo-attractant. Cells were incubated for 24 h (22RV1) and 48 h (LNCaP) before cells on the lower surface of the inserts were fixed in 100% methanol for 1 min at room temperature and stained using DipQuick stain (Fronine, Thermo Scientific, Waltham, MA, USA) according to manufacturer's protocol. The number of migrating cells was totaled from five random fields at ×10 magnification using the Eclipse T*i* microscope (Nikon Instruments Inc., Melville, NY, USA).

### 3D Matrigel™ Cell Culture

Cells were grown between two layers of growth factor-reduced phenol-red free Matrigel^TM^ (BD Biosciences, Becton Drive Franklin Lakes, NJ, USA) in 96-well plates (Corning, NY, USA): bottom layers were formed with 35 μL of Matrigel^TM^/culture medium (3:1; 75%; 7.35 mg/mL of Matrigel^TM^) and polymerized at 37°C for 30 min. Cells were seeded at 1,000 cells/well in Matrigel^TM^/culture medium (1:39, 2.5%; 0.245 mg/mL of Matrigel^TM^) (30, 31). Single cells within the Matrigel^TM^ layers were grown to small spheroidal colonies for 3–4 days, after which the culture medium was switched to CSS with treatments. The colonies were cultured for another 14 days. Insulin and DHT treatments were refreshed every 24 h, and all treatments changed every second day.

### Transwell Invasion Assay

Invasion assays were performed in Modified Boyden Blind Well Chambers with 13 mm diameter 12 μm pore size PVP free polycarbonate filter (Neuro Probe Inc, Gaithersburg, MD, USA). Filters were coated with 50 μL (0.50 mg/mL) of Matrigel^TM^. Pre-treated LNCaP cells (3 × 10^5^ cells/well) were seeded in the upper chamber containing serum-free RPMI-1640 with appropriate treatment and the lower chambers contained RPMI-1640 containing 10% FBS as chemo-attractant. After 24 h, cells were fixed in 100% methanol and stained with DipQuick stain. The total number of invading cells was calculated from counting five representative fields as described above.

### Microarray

LNCaP cells, plated on 10 cm dishes, were incubated in 5% CSS RPMI (Sigma-Aldrich) for 48 h, followed by 24 h incubation in 5% CSS RPMI with 10 nM R1881 or equal volume 20% EtOH (vehicle). Cells were transferred to serum-free RPMI with treatments maintained for 24 h. Ten nanometer insulin was then added in the final 10 h before TriReagent (Sigma-Aldrich) extraction of RNA. All treatments were done in triplicate. A custom designed microarray was used (GEO platform ID GPL17236, Agilent Technologies, Wilmington, DE, USA). Total RNA quality was assessed using the Agilent RNA 6000 Nano Kit (Agilent) on an Agilent 2100 Bioanalyzer and all samples measured with RIN >8. One-color microarray-based gene expression analysis was performed following Agilent's Quick Amp Labeling Kit (Agilent) using 500 ng of total RNA according to manufacturer's instructions. Scanning was performed using Agilent High Resolution Microarray Scanner, and data was processed with the Agilent Feature Extraction 10.5.1 software (Agilent). Data was normalized using the quantile normalization method in LIMMA (32). Results were submitted to GEO (GSE47622). Pathway analysis was performed using Ingenuity Pathway Analysis (Qiagen, San Francisco, USA) against a compiled list of genes associated with epithelial and mesenchymal phenotypes from published literature. Heatmap visualization was performed using GenePattern software (33). Clustering was based on Pearson correlation by pairwise average linkage with data log-transformed.

### RNA Isolation and Quantitative Real Time PCR (qRT-PCR) Analysis

Total RNA was isolated using the Direct-Zol^TM^ RNA Miniprep kit (Zymo Research, Irvine, CA, USA) according to the manufacturer's instructions. Reverse transcription was performed with SuperScript III First-Strand Synthesis system (Invitrogen) and the C1000TM Thermal Cycler (BioRad, Hercules, CA, USA). The targeted gene and primer sequences are described in Supplementary Table 1. Quantitative RT-PCR was performed with a SYBR Green ER qPCR kit (Invitrogen) in a 7900HT Fast Real-time PCR System (Applied Biosciences). The relative expression levels of target genes were normalized to RPL32. Amplification specificity was confirmed by disassociation curve analysis. Each gene was measured in triplicate and the average ^Δ^Ct was taken from the three replicate wells before fold change was calculated using the ^ΔΔ^Ct method.

### Western Blotting

Whole cell protein extracts were lysed with buffer containing 50 mM pH 7.4 HEPES, 150 mM NaCl, 1% Triton-X-100, 1 mM Na_3_VO_4_, 30 mM NaF, 10 mM Na_4_P_2_O_7_, 10 mM EDTA, 0.5 mM AEBSF, and complete protease inhibitor tablet (Roche, Basel, Switzerland) for 20 min on ice. Protein content was determined using the BCA Protein Assay kit (Thermo Scientific, Waltham, MA, USA). Nuclear and cytoplasmic protein extracts were collected using NE-PER®Nuclear and Cytoplasmic Extraction Reagents (Thermo Scientific). Proteins were separated in 10% SDS–polyacrylamide gels and transferred to PVDF membranes. Membranes were blocked in 1% fish skin gelatin (FSG; Sigma-Aldrich) in tris-buffered saline (TBS) or in Odyssey blocking buffer (Thermo Scientific) then incubated with primary antibodies overnight at 4°C. Primary antibodies included anti-Zeb1 (#3396), anti-PI3K (#4257), and phospho-PI3K (#4228), anti-MAPK (#4696) and phospho-MAPK (#4377), anti-PARP (#46D11), and anti-GAPDH (#14C10) from Cell Signaling (Danvers, MA, USA), and anti-FOXC2 (ab55004) (AbCam, Cambridge, UK). After washing with 0.1% TBS-Tween, membranes were incubated with secondary antibodies anti-mouse IgG 800 (926-32213, LI-COR Biosciences, Lincoln, NE, United States), anti-rabbit 700 (926-68072, LI-COR Biosciences), and anti-goat 700 (926-68074, LI-COR Biosciences) diluted 1 in 25,000 in 1% FSG in TBS for 1 h at room temperature and washed. Protein bands were visualized using Odyssey^®^ imaging system (LI-COR Biosciences, Lincoln, NE, USA).

### Gene Expression in Clinical Prostate Cancer Samples

Clinical datasets accessed using Oncomine^TM^ (Compendia Bioscience^TM^, part of Life Technologies^TM^, Ann Arbor, MI, USA) were used to analyze expression of INSR and FOXC2 genes in normal, primary and metastatic prostate cancer tumor tissue. Linear regression and associated statistical analyses were performed using GraphPad Prism (6.00 for Windows, GraphPad Software, La Jolla California USA).

## Results

### Insulin Increases Migration in Androgen Deprived PCa Cells

**Figure 1:**
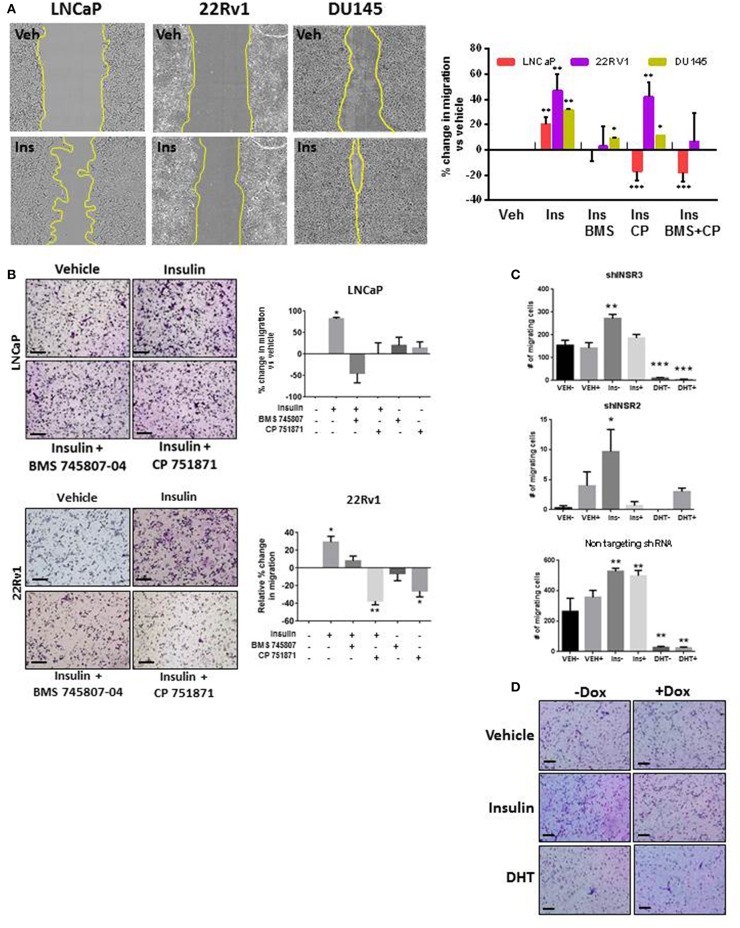
Insulin induced migration in PCa cells is reduced with IR/IGF-1R inhibition. **(A)** Insulin (10 nM) accelerated wound closure in LNCaP, 22RV1, and DU145 cells. Images show wound closure following 24 h insulin treatment, quantitated in the histogram (relative to vehicle). Migration was reduced by the BMS 745807 (10 uM). CP 751871 (5 ug/ml) reduced insulin-induced migration in LNCaP and DU145 cells, but not significantly in 22RV1 cells. **(B)** Insulin also increased transwell migration which was reduced in the presence of BMS 745807 and CP 751871 in LNCaP and 22RV1cells. **(C,D)** LNCaP cells expressing dox-inducible shINSR or a non-targeting shRNA were induced (+) and compared to no dox added (–). The loss of IR expression reduced the number of migrating cells following insulin treatment in shINSR cells but not in non-targeting cells. Ten nanomolar DHT also suppressed cell migration. All scale bars = 100 μm (all graphs *n* = 3, *p < 0.05, **p < 0.01, ***p < 0.001, One-way ANOVA, ± SEM with vehicle control).

To examine the role of insulin in PCa cell migration, we simulated androgen deprivation using charcoal-stripped serum in LNCaP, 22RV1, and DU145 cells for 48 h, then treated cells with 10 nM insulin for a further 48 h as described above. Androgen deprived LNCaP, 22RV1, and DU145 cells displayed increased monolayer migration when treated with insulin (Figure 1A) measured by calculating percent wound confluence compared to vehicle (Figure 1A). Relative wound density over time is shown in Supplementary Figure 1. Insulin can signal through IR homodimers or form hybrid receptors with IGF-1R, dependent on receptor stoichiometry (34). Treatment with BMS 745807 (a pan-insulin/IGF-1R small molecule inhibitor) (35) led to significant suppression of insulin-induced migration in all three cell lines (Figure 1A). CP751871 (IGF-1R-specific blocking monoclonal antibody) (36) blocked insulin-induced migration in LNCaP and DU145 (Figure 1A). Inhibitor doses were based on loss of receptor phosphorylation, assessed by Western blot (Supplementary Figures 2A,B) and treatment with the inhibitors alone did not have any effect on LNCaP cell migration compared to vehicle (Supplementary Figure 2C).

This result was replicated in LNCaP and 22RV1 cells using transwell assays (Figure 1B). We observed increased migration following insulin treatment. Once again this effect was blocked by inhibition of IR signaling. 22RV1 cells showed greater sensitivity to CP751871 between scratch (Figure 1A) and transwell (Figure 1B) migration assays.

To confirm the effects were specifically via activation of the IR, we established LNCaP cell lines with doxycycline (Dox)-inducible expression of shRNA targeting the IR gene (*INSR*). Dox-induced expression of shINSR reduced receptor levels by up to 65% (Supplementary Figures 2D–F) and decreased insulin-induced migration to non-induced cells (Figure 1C) which was not observed in cells expressing a control, non-targeting (NT) shRNA sequence (Figure 1C). Significant inhibition of cell migration was also observed when these cells were treated with 10 nM DHT, regardless of IR knockdown (Figures 1C,D). These data suggest insulin increases migration in AR-dependent and independent cell lines and these effects are specific to the action of insulin/hybrid receptors. These results led us to explore the dependency of this observation on an androgen-low environment.

### AR Regulates Insulin-Induced Migration in PCa Cells

**Figure 2:**
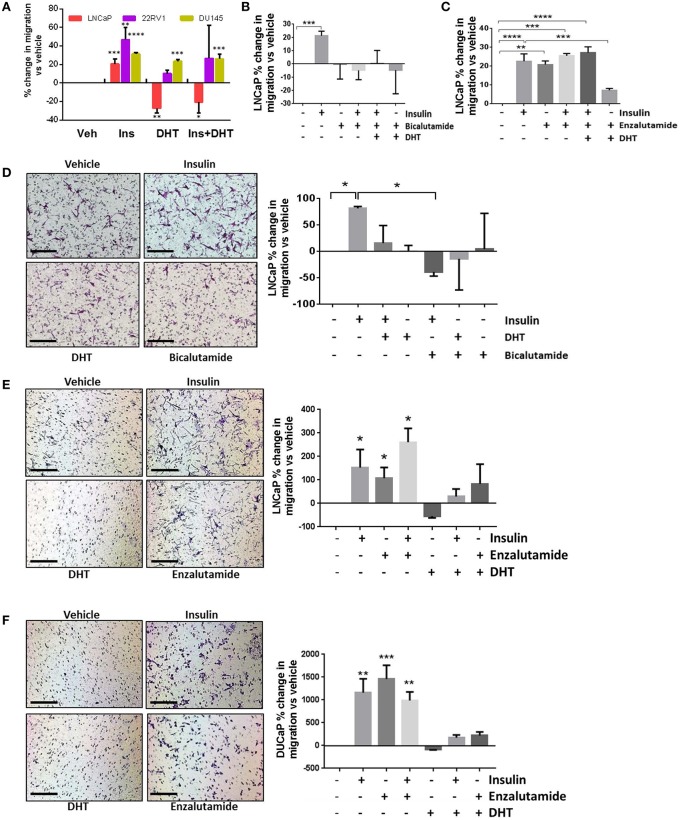
Insulin-induced migration is inhibited by androgens. **(A)** Wound closure assay showed insulin-induced migration was significantly reduced by 10 nM DHT in LNCaP cells. Reduced migration was also observed in 22RV1 cells, but was not statistically significant and DHT had no effect in AR negative DU145 cells. **(B)** In LNCaP cells, bicalutamide (10 μM) inhibited migration, however; **(C)** The addition of enzalutamide (10 μM) induced migration as potently as insulin. **(D)** In transwell migration experiments, insulin induced migration, and both DHT and bicalutamide blocked this effect. **(E)** Enzalutamide, alone and in combination with insulin, significantly increased transwell migration over vehicle. **(F)** In another AR-sensitive cell, DUCAP, insulin, and enzalutamide both significantly increased transwell migration alone and in combination, blocked by DHT (All graphs *n* = 3, *p < 0.05, **p < 0.01, ***p < 0.001, ****p < 0.0001, One-way ANOVA, ± SEM with vehicle as control).

Androgens provide a differentiation pressure to PCa cells which, when removed, results in increased cell motility (7–9). In LNCaP cells, transwell and wound healing migration was lower with the addition of 10 nM DHT (Figures 1C, 2A–C). The addition of DHT to 22RV1 cells, reduced migration but did not ablate it (Figure 2A), consistent with this cell lines reduced sensitivity to DHT and high, ligand-free AR activity and insulin-induced migration in AR negative DU145 cells was unaffected by the addition of DHT (Figure 2A). We used the DHT sensitive cell line, LNCaP, to examine the effects of AR antagonists bicalutamide and enzalutamide. Bicalutamide did not reverse the suppression of insulin-induced migration by DHT (Figure 2B), which may be due in part, to bicalutamide acting as a weak AR agonist in cells which harbor the AR mutation T877A, including LNCaP cells (28, 37). However, enzalutamide (10 μM) antagonized the effects of DHT (Figure 2C) and, in accordance with recent reports (7, 9), induced migration with comparable potency to insulin. The combination of insulin and enzalutamide did not have an additive effect on migration, indicating potential activation of a common pathway, or that the cells may have reached a maximal limit for migration.

In transwell assays, bicalutamide again did not reverse the effects of DHT (Figure 2D) and alone blocked insulin induced migration, which may be due to the activation of AR variants in these cells. The increase in migration by enzalutamide was also observed in transwell assays in LNCaP and an additional androgen sensitive cell line, DuCaP (Figures 2E,F). These results show enzalutamide reversed the inhibition by DHT in insulin treated cells and that insulin driven migration in PCa cells is sensitive to AR activity.

### Insulin Increases Invasion in PCa Cells

**Figure 3:**
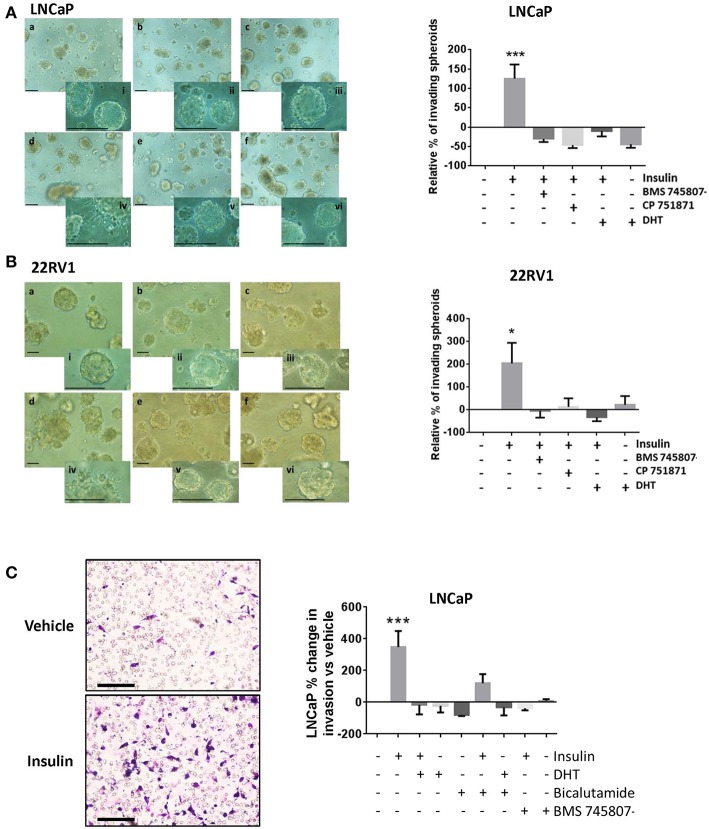
Insulin induced invasion in PCa cells. Quantitation of spheroid growth in 3D Matrigel^TM^ assay following treatment with (a) Vehicle (CSS), (b) insulin + BMS 745807, (c) insulin+DHT (d) insulin, (e) insulin + CP 751871 (f) DHT. Scale bars = 100 μm in image and inset). Insulin (d) promoted spheroid sprouting and invasion in LNCaP cells **(A)** and 22RV1 cells **(B)**, which was blocked with CP 751871 (e) and BMS 745807 (b). Images show degree of sprouting after 3 days of insulin treatment, quantitated in the histogram. Sprouting was not observed in vehicle-treated cells (a) at the same time point. DHT-treated cells showed no evidence of sprouting over this time (f). **(C)** Insulin promoted cell invasion through Matrigel^TM^ coated transwell. The histogram shows addition of DHT completely prevented cell invasion and bicalutamide failed to reverse the inhibitory effect of DHT. BMS 745807 also inhibited insulin induced transwell invasion (All graphs *n* = 3, *p < 0.05, ***p < 0.001, One-way ANOVA, ±SEM with vehicle as control).

To better model the migration and invasion of tumor cells *in situ*, we cultured cells in Matrigel™ to form 3D spheroids and repeated insulin treatments. Insulin increased the rate of invasion from spheroids in androgen deprived LNCaP (Figure 3Ad) and 22RV1 cells (Figure 3Bd) in 3D culture, where single cells were observed migrating away from insulin-treated spheroids after 3 days and invading into the surrounding Matrigel™ by 6 and 5 days, respectively. This increase was blocked by addition of BMS 745807 and CP 751871 in both LNCaP and 22RV1 cell lines (Figures 3Ab,e,Bb,e) and by the presence of DHT (Figures 3Ac,f,Bc,f). Invasion was not observed in vehicle-treated spheroids at the same time point; however, invasive sprouts eventually appeared at days 12–14 post-treatment (data not shown) consistent with previous reports that androgen deprivation is capable of inducing migration and invasion in PCa cells (38). Our results show exposure to insulin significantly accelerates invasion in this low-androgen environment.

Insulin resulted in a dramatic increase in the number of cells undergoing transwell invasion in androgen-deprived LNCaP cells relative to vehicle (Figure 3C). This was blocked by IR inhibitors, DHT and bicalutamide, consistent with our migration data.

### Insulin Increases Expression of EMT and NE Markers in PCa Cells

**Figure 4:**
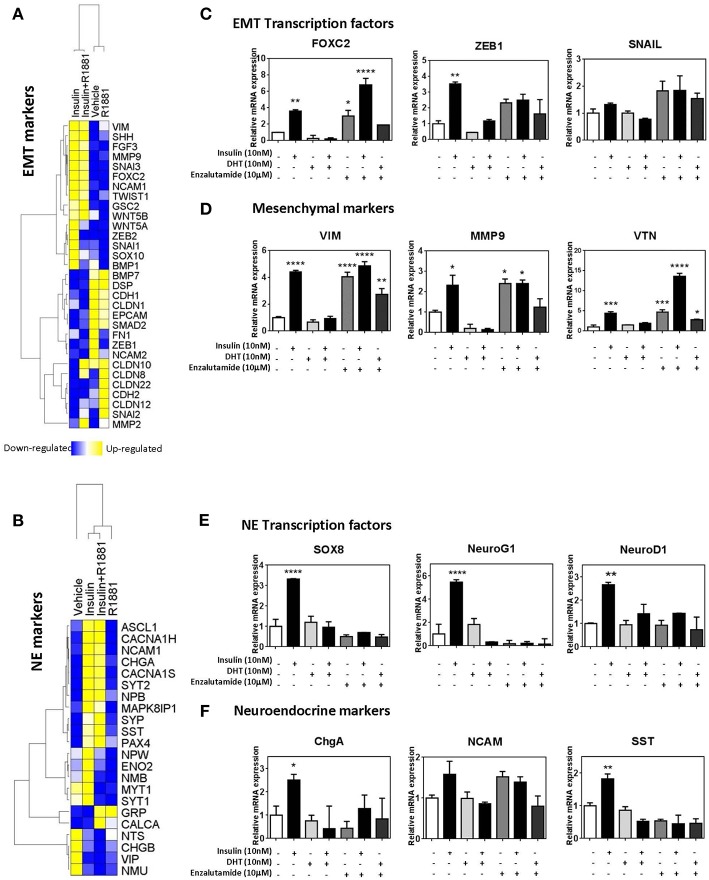
Insulin increases expression of EMT and NE genes in LNCaP cells. Gene expression heatmap of microarray data comparing LNCaP cells treated with 10 nM insulin in the presence or absence of 1 nM R1881 of typical epithelial and mesenchymal genes **(A)** and NE genes **(B)**. qRT-PCR results of LNCaP cells confirm that 48 h insulin treatment up-regulated key EMT associated transcriptional factors, FOXC2, ZEB1, and SNAIL **(C)**, mesenchymal markers, VIM, MMP9 and VTN **(D)**, NE associated transcriptional factors, SOX8, NeuroG1, and NeuroD1 **(E)** and NE markers, ChgA, NCAM, SST **(F)**. 48 h insulin treatment (10 nM) significantly increased levels of transcription factors in LNCaP cells to levels similar to those observed for enzalutamide (10 μM) treatment. DHT (10 nM) suppressed expression of these transcription factors (All graphs *n* = 3, *p < 0.05, **p < 0.01, ***p < 0.001, ****p < 0.0001, One-way ANOVA, ±SEM with vehicle as control).

To identify the molecular mechanisms underlying insulin-enhanced gain of invasion and migration, we examined microarray data from LNCaP cells treated with insulin, in the presence or absence of synthetic androgen R1881. Ingenuity Pathway Analysis identified transcriptomic changes in insulin-treated LNCaP cells consistent with EMT (Figure 4A) and NE transdifferentiation (Figure 4B). These results were validated by qRT-PCR. Insulin increased transcription of hallmark EMT-associated transcription factors, *ZEB1, VIM*, and *FOXC2* (Figure 4C). This increase was not observed in the presence of DHT, however enzalutamide alone modulated gene expression of mesenchymal genes with similar potency to insulin (Figures 4C,D). Insulin increased expression of NE-associated transcription factors *SOX8, NEUROG1*, and *NEUROD1* (Figure 4E) and several markers of NE phenotype including *CHGA* and *SST* (Figure 4F).

Expression of these factors was also examined at 20 h post-insulin treatment (Supplementary Figure 3). Transcription factors for both EMT and NE phenotypes *(FOXC2, ZEB1, SOX8, NEUROG1)* were significantly increased by 20 h, indicating that these transcription factors may be driving subsequent transcription of EMT and NE markers. Markers such as *MMP9, VTN*, and *SST* were also increased with 20 h insulin treatment while other markers such as *VIM* remained unchanged, suggesting *MMP9, VTN*, and *SST* might be under more direct insulin regulation.

**Figure 5:**
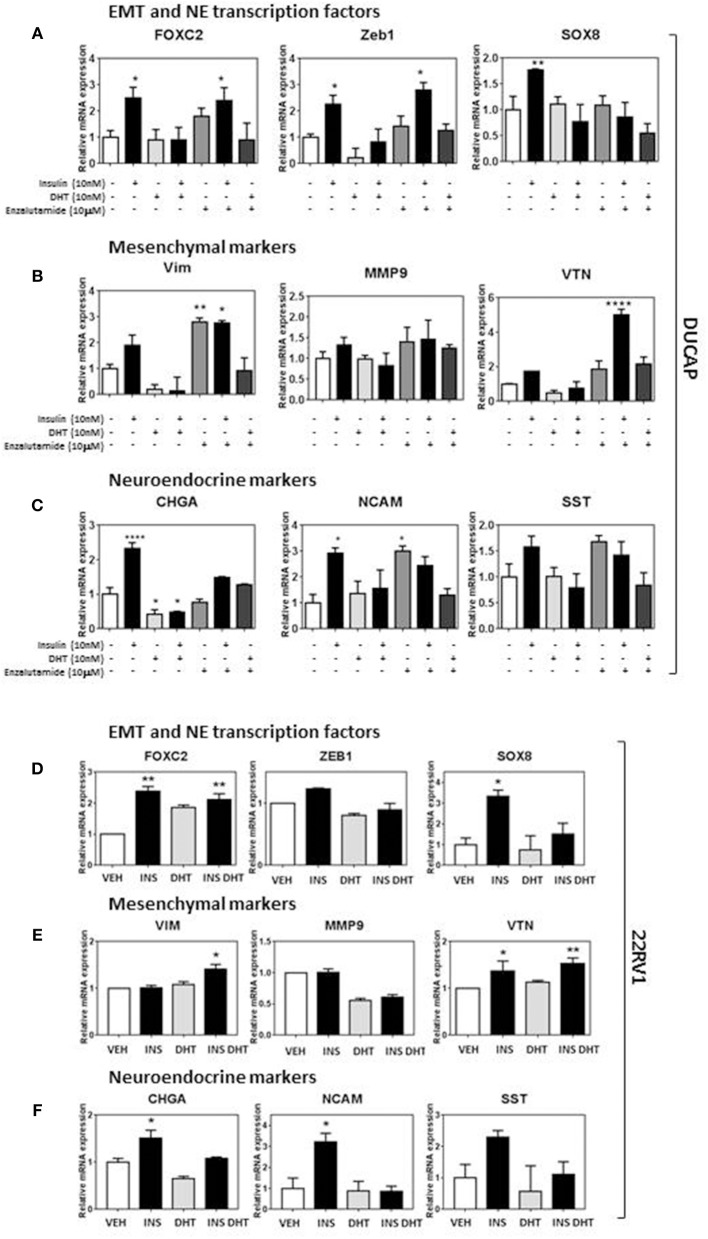
Insulin increases EMT and NE transcriptional profile in DUCaP and 22RV1 cells. To validate the LNCaP observations, 22RV1 and DuCaP cells were cultured under androgen deprived conditions and treated with insulin (10 nM), DHT (10 nM), and enzalutamide (10 μM) (48 h). qRT-PCR shows that levels of both **(A)** EMT and NE transcription factors FOXC2, ZEB1, and SOX8 significantly increase in DuCaP cells with insulin treatment, along with non-significant increases in **(B)** mesenchymal markers VIM and VTN and **(C)** NE markers CHGA, NCAM and SST. **(D)** 22RV1 cells increased transcription factor expression and with **(E)** a muted rise in the mesenchymal transcripts with VTN significantly increased in 22RV1s and no significant change with insulin for VIM and MMP9. **(F)** NE markers CHGA, NCAM, and SST were significantly upregulated with insulin treatment. DUCAP cells replicated LNCaP transcription in response to AR inhibitor enzalutamide for both EMT and NE transcriptional factors, mesenchymal markers and NE markers (All graphs *n* = 3, *p < 0.05, **p < 0.01, ****p < 0.0001, One-way ANOVA, ± SEM with vehicle as control).

Probing the same markers in DuCaP and 22RV1 RNA (Figures 5A–F) revealed similar expression of both EMT and NE associated markers, and reversed in DuCaP with DHT. Some cell-line related differences were observed. *VIM* and *MMP9* levels did not increase in 22RV1 cells in response to insulin (Figure 5E). Additionally, DHT did not block the insulin induction of *FOXC2* and *VIM* in 22RV1 cells, consistent with the reduced androgen sensitivity of this cell line (Figures 5D,E).

Although classical epithelial gene transcripts such as EPCAM and CDH1 were decreased in insulin-treated LNCaPs, compared to DHT-treated cells (Supplementary Figure 4A), these results were not reflected in protein levels which remained stable across all treatments (Supplementary Figures 4B,C). Similar results were seen in 22RV1 (Supplementary Figures 4D,E) and DuCaP (Supplementary Figures 4F,G) cells. Together these data indicate that plasticity mechanisms such as EMT and NE may be activated by insulin in androgen deprived PCa cells, via common transcriptional regulators including *FOXC2*.

### PI3K and MAPK Pathways Signal Insulin Driven Plasticity

**Figure 6:**
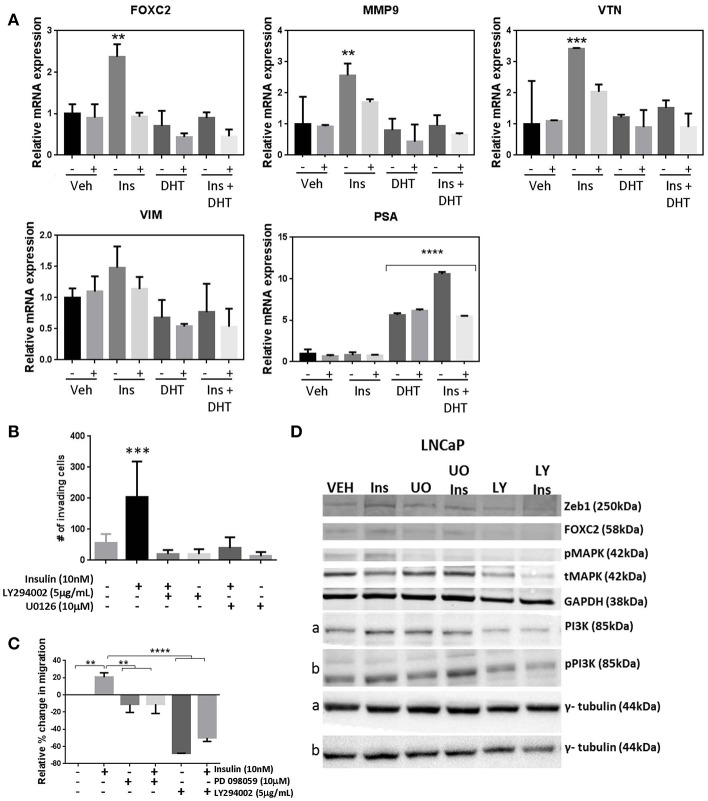
Manipulation of insulin signaling prevents insulin induced migration and expression of plasticity phenotype. **(A)** The shINSR model was used to measure expression of FOXC2, MMP9, VTN, VIM following 10 nM insulin treatment (Ins) with and without 10 nM DHT (10 nM) using 5-day doxycycline (250 ng/mL/day) induced shINSR3 cells. Insulin-induced increases were lost with shINSR induction (± used to denote no/dox induction). DHT inhibits insulin-induction of gene expression. PSA control shows DHT response. **(B)** Inhibitors to PI3K and ERK1/2 reduced both insulin-induced transwell invasion and **(C)** wound-scratch migration in LNCaP cells, and **(D)** prevented insulin induced rise in FOXC2, Zeb1, and phosphorylated MAPK in LNCaP cells. Inhibitor to PI3K (LY294002; LY) reduced total and phosphorylated PI3K in LNCaP, but inhibitors of ERK1/2 of the MAPK signaling pathway (UO0126; UO) increased total and phosphorylated PI3K, suggesting a compensatory pathway (a,b, corresponding tubulin loading) (All graphs *n* = 3, **p < 0.01, ***p < 0.001, ****p < 0.0001, One-way ANOVA, ± SEM with vehicle as control).

Genetic silencing of the IR in LNCaP cells, reduced transcription of EMT markers *FOXC2, MMP9, VTN* following insulin treatment, indicating signaling though the IR drives insulin activated plasticity programs such as EMT (Figure 6A). However, targeting IR is not clinically viable (36). Thus, we tested inhibitors to signaling molecules downstream of IR. The addition of MEK1/2 inhibitors U0126 (Figure 6B) and PD98059 (Figure 6C) prevented insulin induced invasion and migration, and blocked insulin induced phosphorylation of ERK1/2 and increase in FOXC2, Zeb1 protein (Figure 6D; Supplementary Figure 5A). The broad spectrum PI3K inhibitor LY294002 also dramatically reduced insulin induced invasion and migration, and upregulation of FOXC2 and ZEB1 (Figures 6B–D; Supplementary Figure 5A). The crosstalk between PI3K/AKT/mTOR and RAS/RAF/MEK pathways is well-described in PCa, suggesting combination therapy inhibitors targeting both pathways may be required to prevent disease progression (39–41). Thus, these results indicate that PI3K and MEK inhibitors may be useful to counter the effects of insulin in PCa. Together, the data suggests insulin signals through PI3K and MEK pathways downstream of IR or hybrid IR:IGF1R to promote cellular invasion and migration.

### INSR Expression Correlates With FOXC2 in Prostate Tumor Tissue

**Figure 7:**
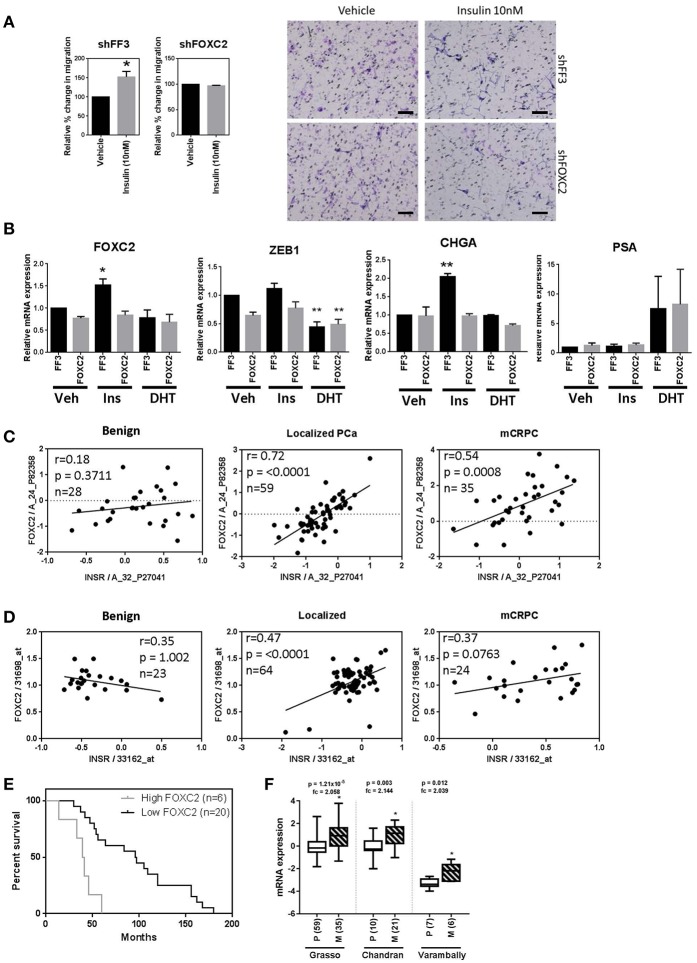
FOXC2 knockdown blocks migration and plasticity phenotype and FOXC2 expression significantly correlates with insulin receptor expression in PCa patient samples. **(A)** LNCaP cells with constitutive FOXC2 knockdown did not migrate through transwell in response to insulin treatment, unlike their control counterparts with scramble knockdown (Scale bars = 100 μm). **(B)** LNCaP cells with constitutive FOXC2 knockdown were also unable to up-regulate several EMT and NE markers in response to insulin (Ins) treatment compared to vehicle (Veh), unlike FF3 control cells. Knockdown of FOXC2 prevented insulin induced rise in Zeb1. Linear regression shows a significant positive association between FOXC2 and INSR in PCa patients with localized and mCRPC tumors in both **(C)** Grasso and **(D)** Yu datasets. **(E)** Kaplan-Meier survival curve shows FOXC2 expression is significantly associated with overall survival in mCRPC patients with wild type AR amplification mutation in Grasso. Median overall survival is significantly reduced by 62% in patients with metastatic tumors expressing high levels of FOXC2 compared with patients with low level of FOXC2 expression **(F)** Box-plots showing FOXC2 mRNA is significantly increased in the metastatic PCa tumors (M) (patient numbers in brackets) compared to primary tumors (P) in three datasets, accessed using Oncomine^TM^ (*p < 0.05, **p < 0.01, One-way ANOVA, ±SEM with vehicle as control).

To determine the effect of key EMT transcription factors on insulin action in PCa cells, LNCaP cells expressing shRNA against *FOXC2* and *ZEB1* were engineered. LNCaP cells with constitutive *FOXC2* knockdown (shFOXC2) did not respond to insulin with increased migration in transwell assays, in contrast to the control cells (shFF3; Figure 7A). Additionally, there was no increase in FOXC2 and CHGA transcripts by insulin in FOXC2 knockdown cells (Figure 7B). To increase sensitivity for Western blot analysis, LNCaP cell lysates were fractionated to nuclear and cytoplasmic fractions. We observed increased nuclear localization of FOXC2 with insulin in the shFF3 control cell line, but no difference between vehicle and insulin in the shFOXC2 cell line. The increase in nuclear levels of Zeb1 by insulin was also diminished in *FOXC2* knockdown cells, as was the rise in total and phosphorylated nuclear ERK1/2 (Supplementary Figure 5B).

In contrast, dox-induced knockdown of *ZEB1* in LNCaP cells dramatically reduced migration in vehicle conditions (androgen-free) making any additional effect of insulin difficult to delineate (Supplementary Figure 6A). QRT-PCR showed that knockdown of Zeb1 with two different vectors in LNCaP cells did not block insulin induction *FOXC2, VTN*, and *MMP9* transcription, thus inhibiting Zeb1 did not prevent insulin induced transcription of mesenchymal genes (Supplementary Figure 6B). Together, these data suggest that FOXC2, rather than Zeb1, drives the insulin induced LNCaP migration.

To investigate the clinical relevance of this discovery, we interrogated the relationship between IR and FOXC2 expression in publicly available transcriptomic databases of clinical tissue through Oncomine^TM^. Expression of *INSR* correlated significantly with *FOXC2* in tumors but not in benign prostate tissue in the Grasso et al. (42) and Yu et al. (43) clinical datasets (Figures 7C,D). No correlation between *INSR* and *ZEB1* was observed (data not shown). This reflects the *in vitro* results where a more striking relationship between insulin signaling and FOXC2, compared to Zeb1, is apparent. Additionally, among patients with metastatic tumors that have amplified wild type AR, increased *FOXC2* mRNA in tumors correlated with significantly reduced median overall survival (Figure 7E). FOXC2 levels were also increased in metastatic PCa tumors compared to primary tumor tissue in three clinical datasets (Figure 7F). These data highlight the potential clinical significance of FOXC2 and the relationship with IR in PCa.

## Discussion

**Figure 8:**
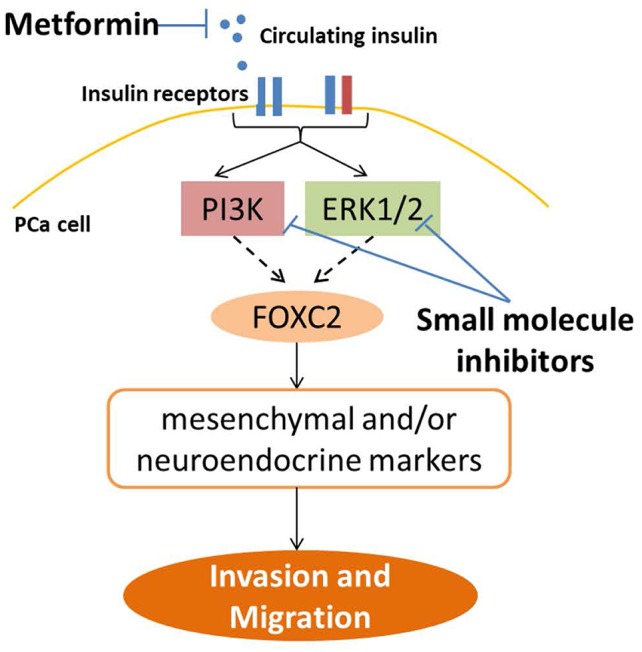
Strategies to inhibit insulin induced PCa invasion and plasticity. This study shows insulin receptors in PCa cells can signal through a combination of PI3K/Akt and RAS/RAF/MAPK pathways, and in LNCaP cells, up-regulate FOXC2 transcription factor, which then activates transcription of a series of EMT and NE markers, to promote gain of cell invasion and migration. This pathway can be blocked top-down by reducing ADT-induced systemic hyperinsulinemia in PCa patients through use of anti-diabetic drug such as metformin, to reduce circulating insulin levels and ligand availability for insulin receptors in PCa cells. Alternatively, signaling may also be targeted downstream of the insulin receptors by targeting PI3K and MAPK pathways with small molecule inhibitors. Direct inhibition of insulin receptors, either IR or IGF1R, leads to worsening of insulin resistance and hyperinsulinemia in patients and are not viable for clinical use in PCa (36).

This study is the first of its kind to demonstrate insulin directly regulating invasion and migration in PCa cells. Insulin has previously been shown to increase invasion in endometrial and pancreatic cancer cells (44, 45), but these studies did not explore the transcriptional and translational profiles of this migration. In our study, insulin augments adaptive plasticity via an independent mechanism to androgen deprivation, without further changing levels of epithelial proteins. We also showed, for the first time, that insulin upregulates NE markers in PCa *in vitro*. Furthermore, upregulation of IR is associated with an invasive phenotype and in patient tumors implies a significantly reduced prognosis (46). Thus, in the absence of androgens, insulin modulates the molecular profile of cancer cells consistent with a more invasive phenotype. Our results suggest this occurs through PI3K/Akt and Ras/Raf/MAPK signaling and that transcription factor FOXC2 may play a key role (Figure 8). Previous studies support that Ras/Raf/MAPK (47) and PI3K/Akt/mTOR (48) pathways can regulate NE phenotype in PCa. DHT can increase migration and invasion (5, 6), influenced by duration of experiments, feedback via the AR axis, method of measuring migration and suppression of cell growth (49, 50).

Insulin has been shown to regulate FOXC2 in human endothelial cells (51) and mouse adipocytes (52) but this is the first time that insulin regulation of FOXC2 has been demonstrated in PCa cells. FOXC2 is reported to regulate both EMT and NE plasticity mechanisms in PCa (53), with downstream activation of Zeb1, as reflected in our study (Figures 4–6 and Supplementary Figure 5). The observation that FOXC2 activates a mesenchymal phenotype without changing epithelial markers is also consistent with previous studies in breast and PCa (30, 53). Such a partial EMT (54), where cells retain epithelial characteristics, while gaining mesenchymal characteristics such as MMP9 expression (55) as observed in our study, has been associated with the metastatic phenotype (56). Elevated MMP9 has been reported to increase in both cell migration in either sheet, clusters or chains, known as collective migration and single cell migration. Thus, insulin may increase collective migration in PCa cells.

FOXC2 and insulin display positive feedback mechanisms in adipose tissue where FOXC2 is increased downstream of insulin signaling and in turn further increases the sensitivity of the tissue to insulin (57–59). Whether FOXC2 mediates similar effects in PCa tumors requires further investigation. A recent study showed that *in vivo* inhibition of p38MAPK reduced the effects of FOXC2 in PCa (53). While we did not investigate p38MAPK inhibitors, we report that inhibition of ERK1/2 (p42MAPK) by MEK inhibitors leads to reduction in FOXC2 as well as insulin driven invasion and migration. FOXC2 is a phosphoprotein (60), phosphorylated by both p38MAPK (53) and ERK1/2 (61). Knockdown of FOXC2 reduced ERK1/2 levels, indicating FOXC2 may directly regulate the RAS/MAPK/ERK1/2 pathway, as previously described (62). Further studies are needed to unveil genomic and non-genomic targets of FOXC2 as well as its regulation of and by the RAS/MAPK/ERK pathway. Importantly, ERK1/2 inhibitors may be effective at inhibiting FOXC2 action in PCa similarly to p38MAPK inhibitors (53).

The differences in insulin responsiveness among PCa cell lines could be due to differential expression of IR and IGF1R. This could result in differential downstream signaling as different levels of IGF1R or IR isoforms result in differences in hybrid receptor formation or increased IR-A driving more migratory pathways than IR-B (63). The PTEN status of PCa cells may also contribute to differences in migration response, where PTEN mutations prime cells for EMT and invasion through additional activation of RAS/RAF/MEK pathway, without which significant metastatic burden is not achieved (64). Unlike both LNCaP and DU145 cells, 22RV1 cells, with wild type PTEN, do not have a basal activated PI3K pathway, which may alter signaling networks downstream of IR in these cells and result in a muted EMT response.

These *in vitro* results suggest ADT-induced hyperinsulinemia can impact on cancer progression and potentially aide in metastasis-related processes. Hyperinsulinemia has been specifically reported to promote breast cancer metastasis to the lung in an elegant mouse model of non-obese insulin resistance (65). Our findings highlight the need for monitoring insulin levels in PCa patients receiving ADT, who will predictably develop hyperinsulinemia, and build a strong case for targeting insulin and downstream activated pathways.

IR inhibitors are not clinically useful, due to their potential to cause hyperglycemia. Inhibitors to IGF-1R, which target hybrid receptors, also have undesirable metabolic effects (36), as well as induce resistance mechanisms by the tumor via up-regulation of IR (66). Our results indicate that PI3K and MAPK inhibitors may be useful in PCa patients who present with or develop ADT induced hyperinsulinemia, with recent studies advocating the use of inhibitors combinations to target multiple pathways (39–41, 64, 67) (Figure 8). Further studies investigating the protein interactions upstream of insulin induced transcription in PCa cells may produce useful targets for inhibition of this specific pathway. Use of small molecule inhibitors to PI3K and MAPK have not shown the promised efficacy in clinical trials and can additionally induce hyperinsulinaemia (68, 69) however, recent animal studies indicate that this can be improved by reducing circulating insulin levels, especially for inhibitors of PI3K p110α subunit (68). To this end, ligand control may be the appropriate adjuvant intervention for patients. Hyperinsulinemia may be targeted by diet alterations (68) or via re-positioning diabetes drugs such as the insulin-sensitizing drug metformin as adjuvant ADT therapy, to reduce circulating ligand levels (70) (Figure 8). Although further work is required to characterize recommended methods of combating hyperinsulinaemia (68, 71), recent literature suggests metformin may improve both quality of life and overall survival by reducing insulin resistance and hyperinsulinemia and associated negative impacts on PCa progression, but may also have direct anti-cancer effects by inhibiting PCa proliferation (70). Thus, further studies to understand the molecular mechanisms of insulin-lowering drugs and diet in PCa are needed.

This study proved that insulin can directly increase migration and invasion in androgen deprived PCa cells *in vitro*. Mechanistically, this study demonstrated for the first time that signaling through the IR and downstream PI3K and MAPK pathways, insulin can activate EMT and NE phenotype and there is significant clinical correlation between IR and the EMT transcription factor FOXC2 in PCa tumors, associated with decreased survival.

## Ethics Statement

This study used commercially available human cell lines and the patient tumor data in Figure 7 was accessed via Oncomine^TM^ and did not require additional ethical approval from our institution.

## Author Contributions

Specifically, microarray and bioinformatics analyses (AL, ML, JG, and PS), invasion and migration assays (PS, JG, NS, and EW), QRT-PCR and western blot analyses (PS, JG, WL, and AS), and knock-down models (BH, PS, JG, and WL). Drafting manuscript was performed by PS, JG, BH, EW, CN, and ML. All authors were involved in revising it critically for important intellectual content and have given final approval of the version to be published. Each author has participated sufficiently in the work to take public responsibility for appropriate portions of the content and are accountable for the accuracy or integrity of all parts of the work. All authors made substantial contributions to conception and design, acquisition of data, analysis, and interpretation of data.

### Conflict of Interest Statement

The authors declare that the research was conducted in the absence of any commercial or financial relationships that could be construed as a potential conflict of interest.

## Abbreviations

3D: three-dimensional
ADT: androgen deprivation therapy
Akt: alpha serine/threonine-protein kinase
APCRC-Q: Australian Prostate Cancer Research Cancer – Queensland
AR: androgen receptor
cDNA: complimentary DNA
CO2: carbon dioxide
CRPC: castrate resistant prostate cancer
CSS: charcoal stripped serum
DHT: dihydrotestosterone
DMEM: Dulbecco's modified eagle medium
DNA: deoxyribonucleic acid
Dox: doxycycline hyclate
DU145: dura mater 145 cell line
DuCaP: dura mater cancer of prostate cell line
EDTA: ethylenediaminetetraacetic acid
EMT: epithelial to mesenchymal transition
EPCAM: epithelial cell adhesion molecule
ERG: ETS related gene
ERK: extracellular signal regulated kinase
FBS: fetal bovine serum
FOXC2: forkhead box protein C2
GAPDH: glyceraldehyde phosphate dehydrogenase
H&E: hematoxylin and eosin
IGF1R: insulin-like growth factor receptor 1
INSR-A: insulin receptor isoform A gene
INSR-B: insulin receptor isoform B gene
IR: insulin receptor protein
IR-A: insulin receptor isoform A protein
IR-B: insulin receptor isoform B protein
LNCaP: lymph node cancer of prostate cell line
MAPK: mitogen activated protein kinase
mCRPC: metastatic castrate resistant prostate cancer
MDV3100: enzalutamide
MEK: mitogen-activated protein kinase kinase
MMC: mitomycin c
MMP9: matrix metalloprotease 9
mRNA: messenger RNA
NE: neuroendocrine
NEUROG1: neurogenin 1
NEPC: neuroendocrine prostate cancer
OS: overall survival
PBS: phosphate buffered saline
PBST: phosphate buffered saline with Tween-20
PCa: prostate cancer
PCR: polymerase chain reaction
PI3K: phosphoinositide 3-kinase
PSA: prostate specific antigen
qRT-PCR: quantitative real time polymerase chain reaction
Raf, proto-Oncogene: Serine/Threonine Kinase
Ras, proto-Oncogene: GTPase
RNA: ribonucleic acid
RNAi: inducible RNA
RPL32: 60S ribosomal protein 32
RPMI-1640: Roswell Park Memorial Institute medium 1640
SDS-PAGE: sodium dodecyl sulfate polyacrylamide gel electrophoresis
SEM: standard error mean
shRNA: short hairpin ribonucleic acid
shRNAmir: microRNA-adapted shRNA
SNAIL: Snail Family Zinc Finger 1
SOX8: sex determining region Y box 8
SST: somatostain
SYP: synaptophysin
VIM: vimentin
VTN: vitronectin
ZEB1: zinc finger E-box-binding homeobox 1.
