# Supplementary material for: Insulin Enhances Migration and Invasion in Prostate Cancer Cells by Up-Regulation of FOXC2
Source: Front Endocrinol (Lausanne). 2019 Jul 17;10:481. doi: 10.3389/fendo.2019.00481 (PMC6652804; doi:10.3389/fendo.2019.00481)
Supplement: Supplementary file 1 [file Image_1.pdf]

Supplementary Figure 1

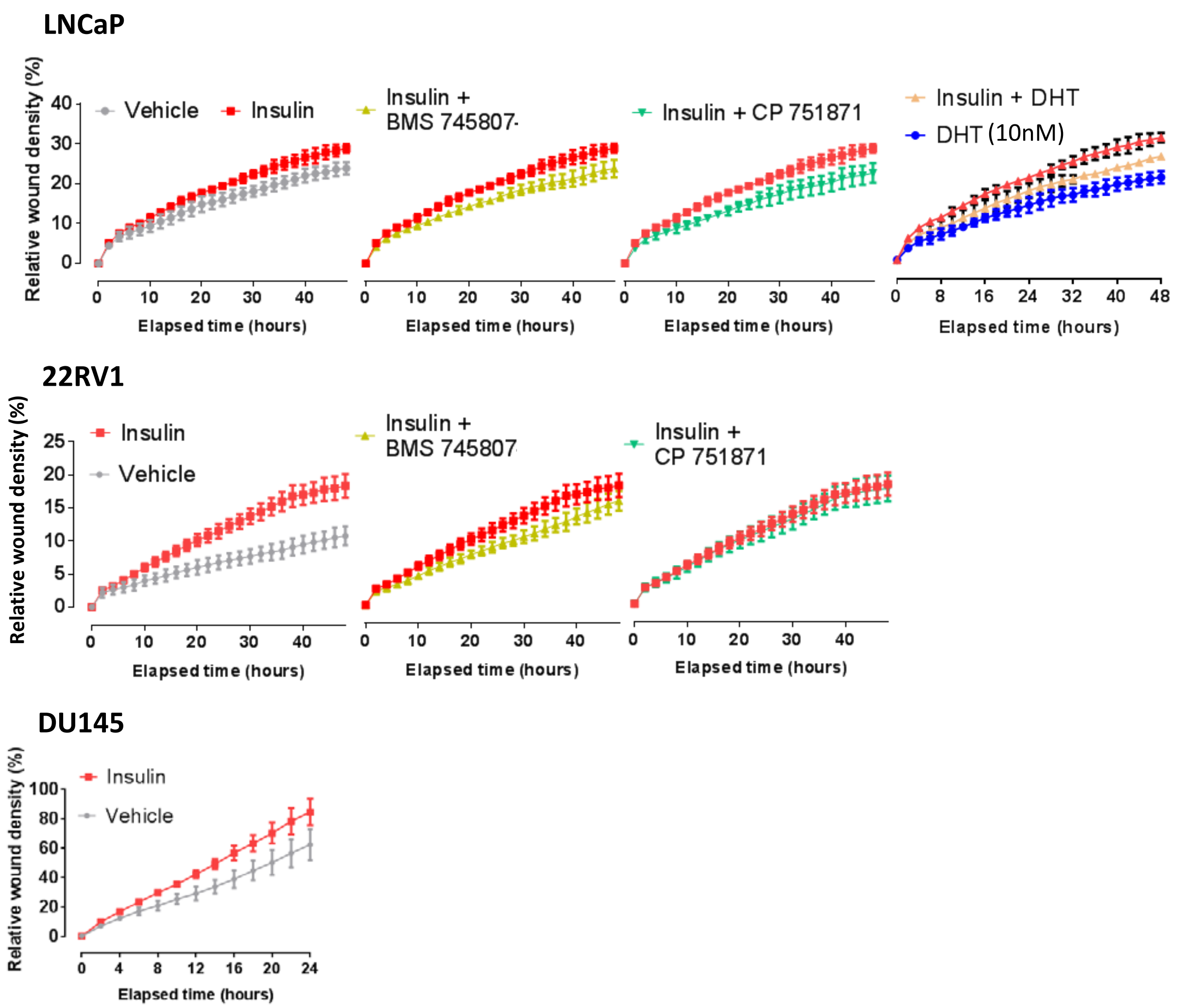

Supplementary figure 1: Insulin induced migration in PCa cells. In androgen deprived conditions wound confluency calculated from real time cell imaging showed insulin (10nM) accelerated wound closure in LNCaP, 22RV1 and DU145. The addition of 10µM pan insulin/IGF1R inhibitor BMS 745807 reduced the effects of insulin. The monoclonal antibody to IGF1R, CP 751871 (5µg/ml), reduced insulin-induced migration in LNCaP and DU145 cells, but had insignificant impact on insulin-induced migration in 22RV1 cells (n=3, mean ± SEM).
