# Supplementary material for: Insulin Enhances Migration and Invasion in Prostate Cancer Cells by Up-Regulation of FOXC2
Source: Front Endocrinol (Lausanne). 2019 Jul 17;10:481. doi: 10.3389/fendo.2019.00481 (PMC6652804; doi:10.3389/fendo.2019.00481)
Supplement: Supplementary file 2 [file Image_2.pdf]

Supplementary Figure 2

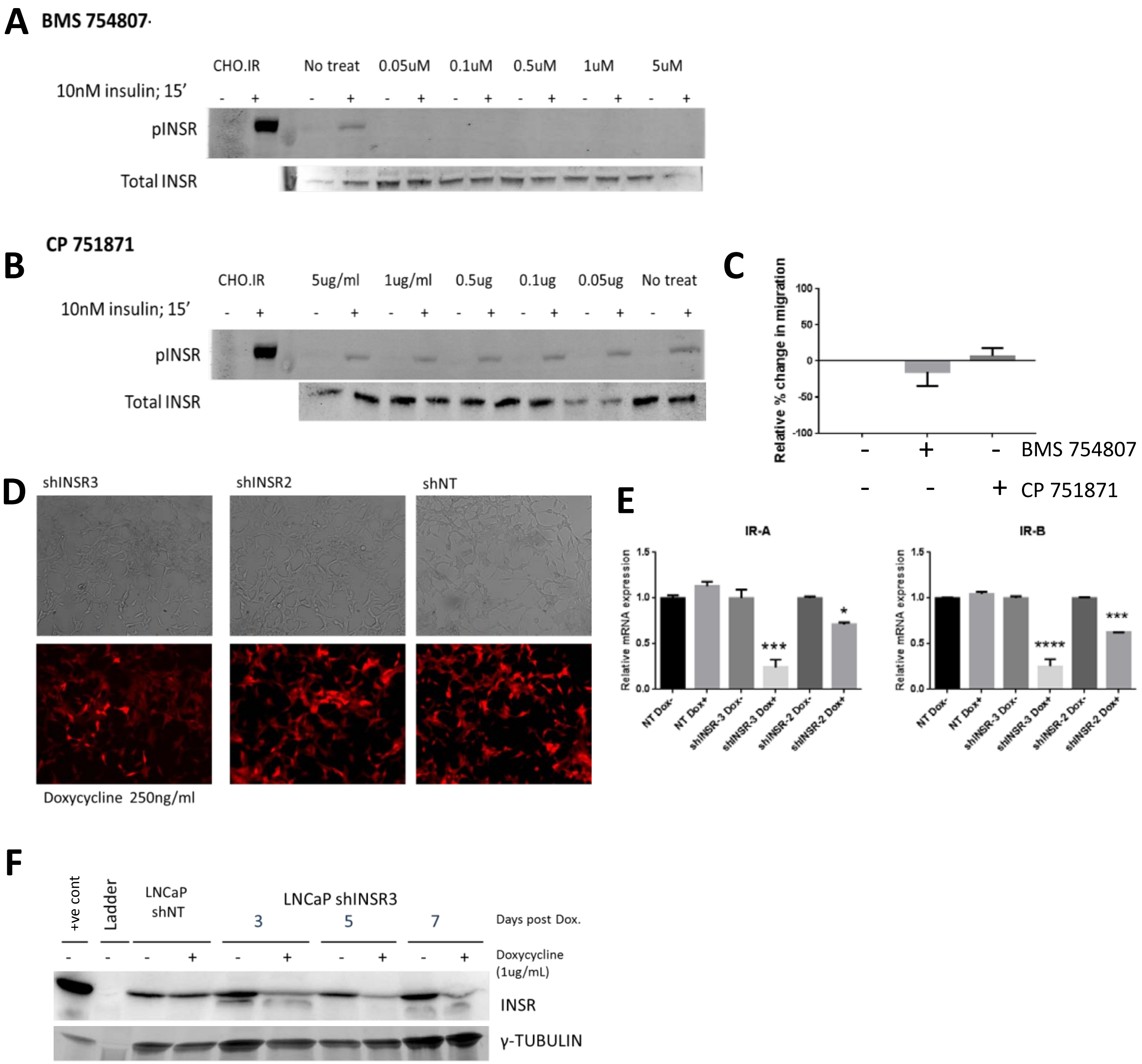

Supplementary figure S2: Optimization of insulin receptor (INSR) inhibitor concentrations. (A) BMS 754807 prevented phosphorylation of insulin receptor at 0.05µM in LNCaP whole cell lysates. (B) CP751871 reduced insulin receptor phosphorylation (pINSR) at 5µg/mL in LNCaP whole cell lysates. Lysates from Chinese hamster ovary cells overexpressing insulin receptor (CHO.IR) were used as the positive antibody control. (C) BMS 745807 or CP751871 did not produce significantly different migration compared to vehicle control treated LNCaP cells in incucyte wound-scratch assays (n=3). LNCaP cells transduced with two separate doxycycline (dox)-inducible shRNA sequences against the insulin receptor (INSR) (shINSR2 and shINSR3 cells) and control cells with non-targeting shRNA (shNT cells) were produced with red fluorescent protein (RFP) as a reporter for doxycycline action. Cells were cultured under normal conditions and treated with 250ng/mL daily top up of doxycycline and 250ng/mL of puromycin every other day to maintain selection pressure. (D) Brightfield and fluorescent images show expression of RFP in cells treated with doxycycline and resulted in decreased INSR RNA (E) and IR protein (F) after 3, 5 and 7 day dox induction compared to non-targeting control (NT).
