# Supplementary material for: Insulin Enhances Migration and Invasion in Prostate Cancer Cells by Up-Regulation of FOXC2
Source: Front Endocrinol (Lausanne). 2019 Jul 17;10:481. doi: 10.3389/fendo.2019.00481 (PMC6652804; doi:10.3389/fendo.2019.00481)
Supplement: Supplementary file 3 [file Image_3.pdf]

Supplementary Figure 3

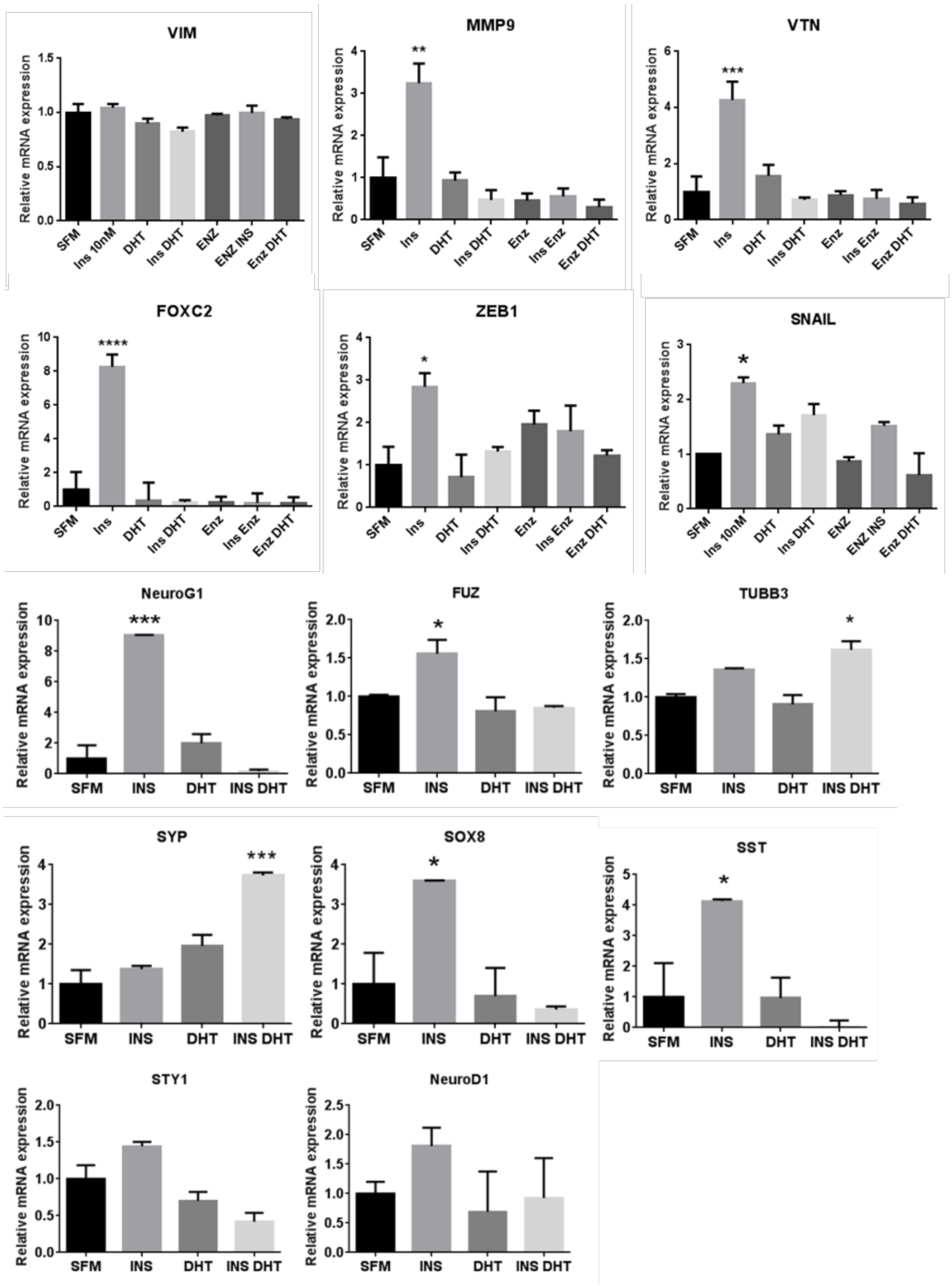

Supplementary figure 3: Early transcriptional response to insulin in LNCaP cells. In androgen deprived LNCaP cells, 20h of insulin (10nM) treatment increased expression of several EMT associated transcripts such as FOXC2, Zeb1, Snail, MMP9 and VTN, as well as NE associated transcripts such as NEUROG1, FUZ, SOX8 and SST. Treatments included serum free media (SFM), insulin (INS) and dihydrotestosterone (DHT). With the exception of SYP transcript up-regulation with insulin (10nM) and DHT (10nM) co-treatment, there was no significant concomitant changes in the other molecules investigated or with other treatments such as enzalutamide at this time point. (n=3, *p<0.05, **p<0.01, ***p<0.001, One-way ANOVA, ± SEM)
