# Supplementary material for: Insulin Enhances Migration and Invasion in Prostate Cancer Cells by Up-Regulation of FOXC2
Source: Front Endocrinol (Lausanne). 2019 Jul 17;10:481. doi: 10.3389/fendo.2019.00481 (PMC6652804; doi:10.3389/fendo.2019.00481)
Supplement: Supplementary file 4 [file Image_4.pdf]

# Supplementary Figure 4

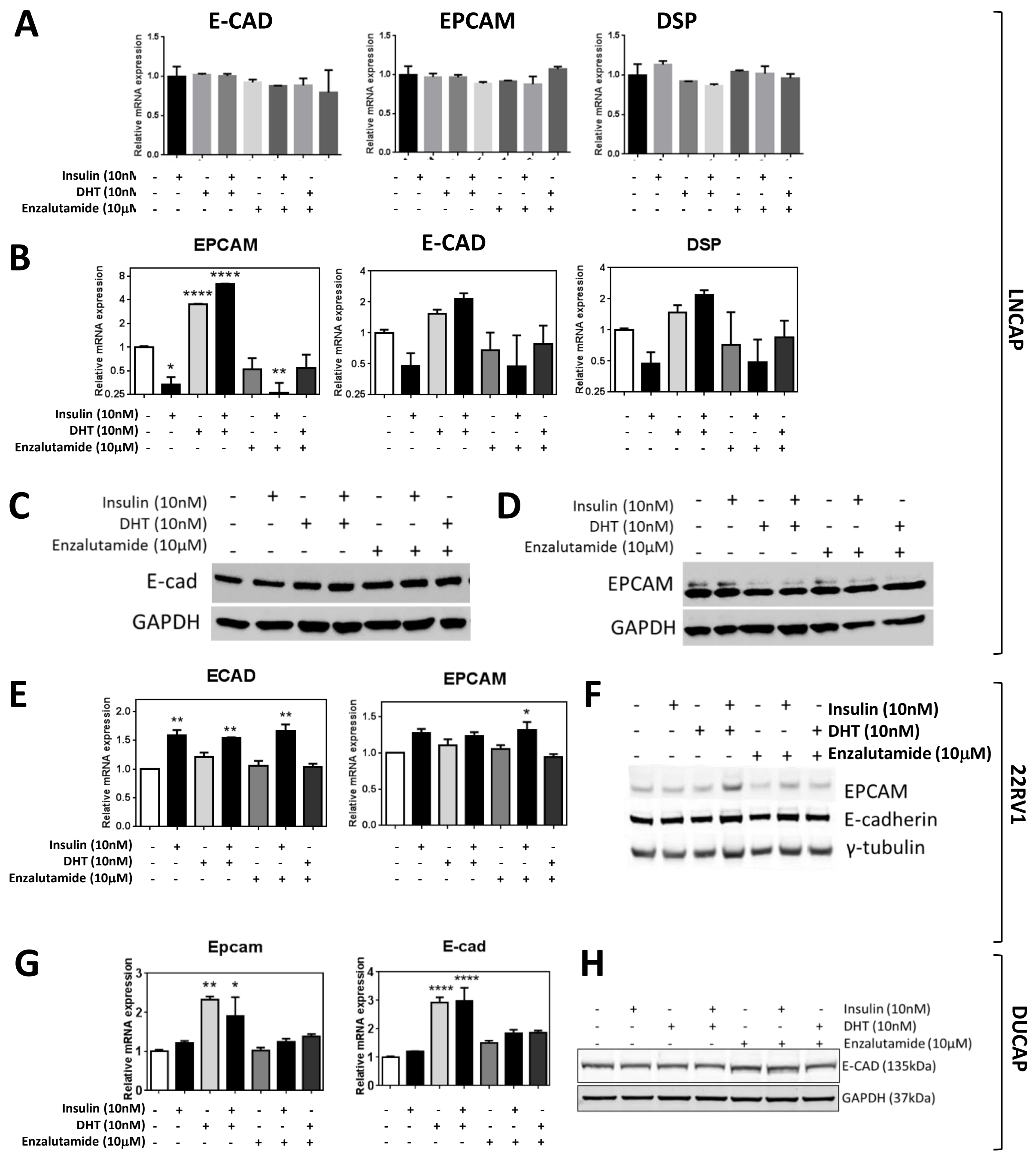


**Supplementary figure 4: No change in protein levels of epithelial markers across different cell lines**. (A) No change was observed in transcription of epithelial markers in LNCaPs treated with insulin (10nM, DHT (10nM) and enzalutamide (10µM) for 20hr. (B) After 48h of treatment of androgen deprived cells, insulin (10nM) induced transcriptional downregulation of epithelial markers EPCAM, E-cad and DSP in LNCaP cells, which were up-regulated by addition of DHT (10nM). AR inhibition by enzalutamide (10µM) reversed the effects of DHT and mirrored those of insulin. However, protein levels of both E-cad (C) and EPCAM (D) remained stable across the treatments. (E) Insulin increased transcripts of epithelial markers in 22RV1 cells, but did not affect protein levels (F). DUCaP data replicates LNCaP results, where EPCAM and E-cad transcripts are increased in response to DHT (G), but protein levels remain stable across different cell lines (H) (n=3, *p<0.05, **p<0.01, ****p<0.0001, One-way ANOVA, ± SEM)
