# Supplementary material for: Insulin Enhances Migration and Invasion in Prostate Cancer Cells by Up-Regulation of FOXC2
Source: Front Endocrinol (Lausanne). 2019 Jul 17;10:481. doi: 10.3389/fendo.2019.00481 (PMC6652804; doi:10.3389/fendo.2019.00481)
Supplement: Supplementary file 5 [file Image_5.pdf]

Supplementary Figure 5

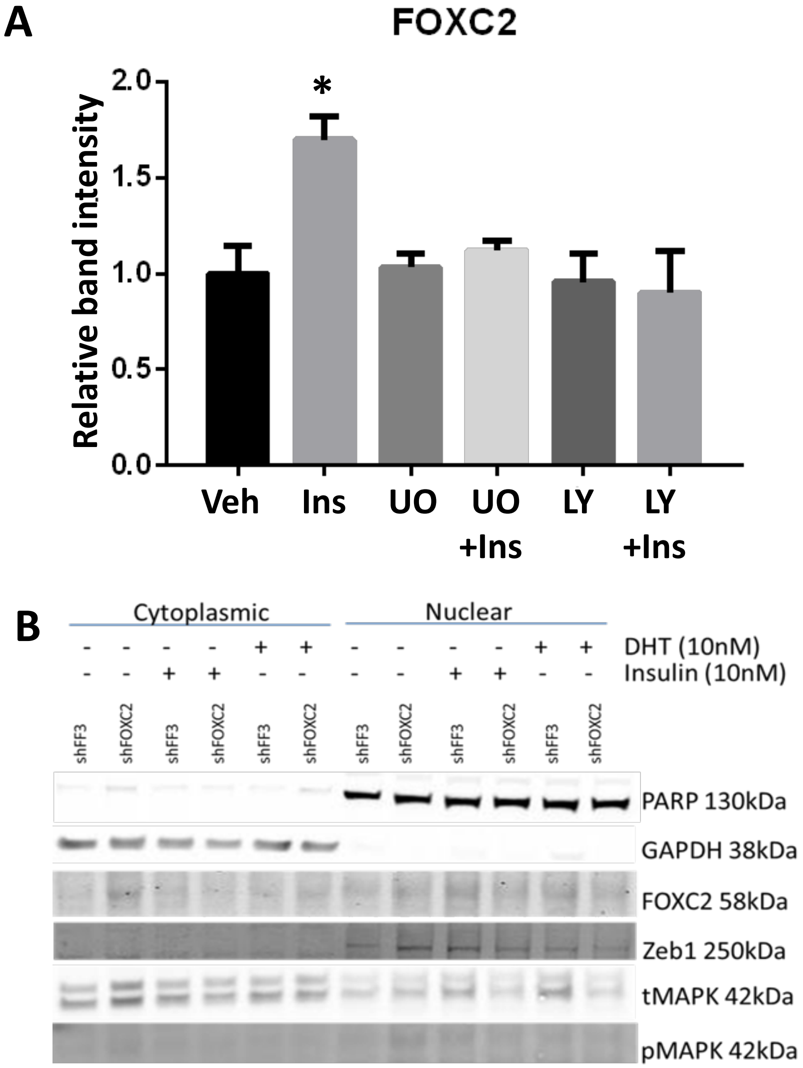

Supplementary figure 5: (A) Quantification of FOXC2 protein levels in whole cell lysates in LNCaP cells treated with insulin (10nM), UO126 (10μM), and LY294002 (5μg/mL) show insulin driven rise in FOXC2 levels is decreased by the PI3K and MAPK inhibitors (n=3, *p<0.05, One-way ANOVA, ± SEM) **(B)** Lysates from LNCaP cells with shRNA against FOXC2 (shFOXC2) and control cells (shFF3) treated with insulin (10nM) and DHT (10nm) for 24h and fractionated into nuclear and cytoplasmic fractions. GAPDH used as cytoplasmic marker and PARP used as nuclear marker. Knockdown of FOXC2 reduced nuclear levels of FOXC2 and Zeb1, and reduced total and phosphorylated nuclear MAPK.
