# Supplementary material for: Insulin Enhances Migration and Invasion in Prostate Cancer Cells by Up-Regulation of FOXC2
Source: Front Endocrinol (Lausanne). 2019 Jul 17;10:481. doi: 10.3389/fendo.2019.00481 (PMC6652804; doi:10.3389/fendo.2019.00481)
Supplement: Supplementary file 6 [file Image_6.pdf]

Supplementary Figure 6

A

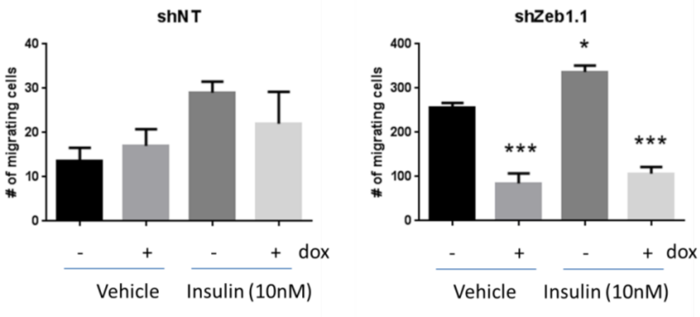

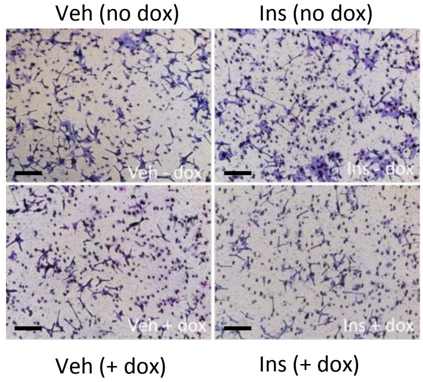

B

<u>LNCaP shNT</u>

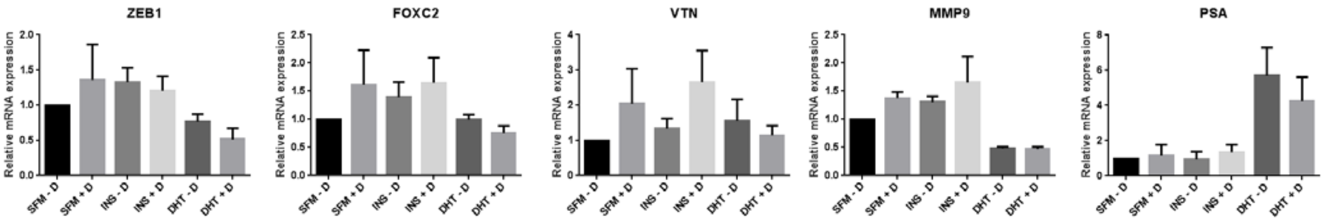

<u>LNCaP shZeb1.1</u>

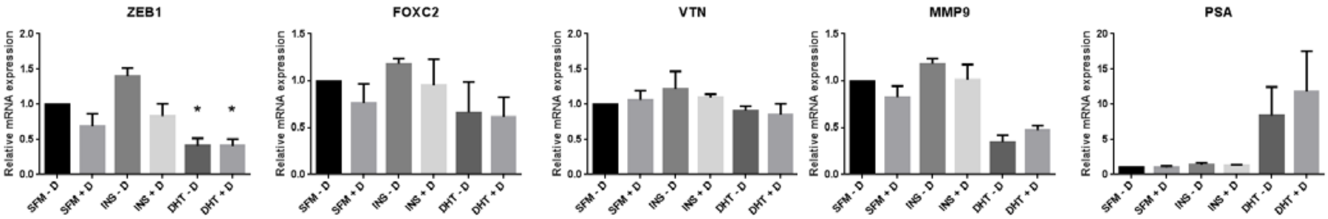

<u>LNCaP shZeb1.2</u>

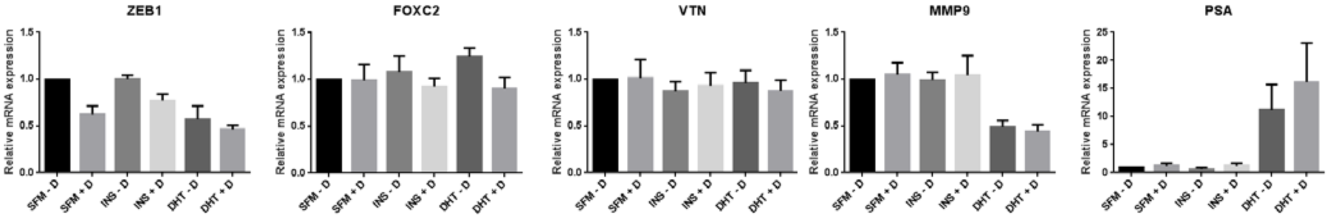

Supplementary figure 6: Knockdown of Zeb1 prevents migration. (A) LNCaP cells with inducible Zeb1 knockdown (shZeb1) were unable to migrate through a transwell in response to 40h insulin treatment follwing 3 days doxycycline induction (250ng/ml), unlike their control counterparts (shNT) (scale bars = 100μm). (B) Reduction in Zeb1 transcripts were observed for the two LNCaP knockdown cells lines, that were not observed for in LNCaP shNT, including with insulin treatment. However, insulin treatment did not significantly increase FOXC2 in response to insulin treatment in the Zeb1 knockdown cells. (n=3, *p<0.05, ***p<0.001, One-way ANOVA, ± SEM)
