# Supplementary material for: Insulin Enhances Migration and Invasion in Prostate Cancer Cells by Up-Regulation of FOXC2
Source: Front Endocrinol (Lausanne). 2019 Jul 17;10:481. doi: 10.3389/fendo.2019.00481 (PMC6652804; doi:10.3389/fendo.2019.00481)
Supplement: Supplementary file 7 [file Image_7.pdf]

Supplementary Figure 7

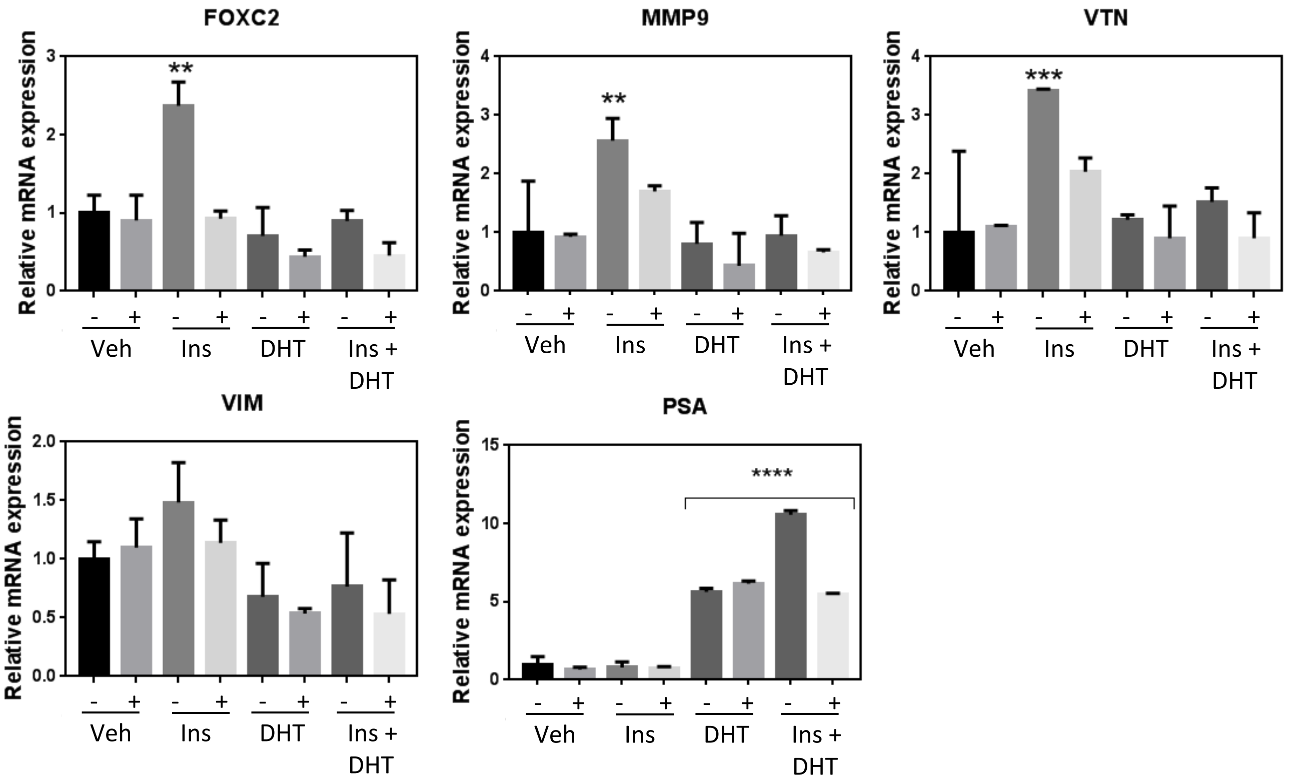

Supplementary figure 7: **Insulin induced EMT blocked by IR knockdown.** Expression of FOXC2 and other mesenchymal markers following insulin (Ins, 10nM) +/- DHT (10nM) treatment using 5-day doxycycline (250ng/mL/day) induced shINSR3 cells. Insulin-induced increases are muted with shINSR induction. DHT inhibits insulin-induction. PSA control for DHT response. (n=3, **p<0.01, ***p<0.001, One-way ANOVA, ± SEM)
