# Supplementary material for: Insulin Enhances Migration and Invasion in Prostate Cancer Cells by Up-Regulation of FOXC2
Source: Front Endocrinol (Lausanne). 2019 Jul 17;10:481. doi: 10.3389/fendo.2019.00481 (PMC6652804; doi:10.3389/fendo.2019.00481)
Supplement: Supplementary file 8 [file Table_1.pdf]

Supplementary Table 1

Gene	Forward primer (5'-3')	Reverse primer (5'-3')
RPL32	CCCCTTGTGAAGCCCAAGA	GACTGGTGCCGGATGAACTT
FOXC2	GCCTAAGGACCTGGTGAAGC	TTGACGAAGCACTCGTTGAG
ZEB1	CAACTACGGTCAGCCC	GCGGTGTAGAATCAGAGTC
SNAIL	CCTCCCTGTCAGATGAGGAC	CCAGGCTGAGGTATTCCTTG
VIM	ACCAGCTAACCAACGACAAAG	GCTTCCTCTCTCTGAAGCAT
MMP9	CTGGGGAAGGAGCCAGTTTG	AAAAACAAAGGTGAGAAGAGAGGG
VTN	GTGGCTGTCCTTGTTCTCCAG	AAGACACTCTGGATGGGTTCAC
CDH1	GAACGCATTGCCACATACAC	ATTCGGGCTTGTTGTCATTC
EPCAM	TGCTCAAAGCTGGCTGCCAAA	GTGCCGTTGCACTGCTTGGC
DSP	AAATTACCCTCCCAGCACCAG	TCCACACTCTGAAACTAAAGGAGA
PSA	AGTGCGAGAAGCATTCCCAAC	CCAGCAAGATCACGCTTTTGTT
NEUROG1	GCCTTTCTATCTGTCCGTCG	GTCTGGCACAGTCTTCCTC
NEUROD1	ACTCCAAGACCCAGAAACTGTC	ACTGGTAGGAGTAGGGATGCAC
SOX8	CCACAAGAGTGCCCCGTC	CTGCAGGAACCGTAGTCGG
FUZ	ACAGACATGGGGCTGTTAGC	AAGCACAGAGGGGAGAGGG
TUBB3	AGCAAGGTGCGTGAGGAGTAT	TGGACAGCGTGGCGTTGTAG
SYP	GGCTCTGGCCACCTACATCTTC	CCGATGAGCTAACTAGCCACATGA
SST	CTGAACCCAACCAGACGGAG	AGCCGGGTTTGAGTTAGCAG
STY1	AACATGGGGTTGGCTGTTT	CGGCAGACGGTTATTTTCCT
CHGA	TCCCTGTGAACAGCCCTATGAATAA	AAAGTGTGTCGGAGATGACCTCAA
NCAM	TGCGACCATCCACCTCAAAG	CCAGAGTCTTTTCTTCGCTGC
INSR-A	CTGCACAACGTGGTTTTCGT	ACGGCCACCGCACATTC
INSR-B	CGTCCCCAGAAAAACCTCTTC	ACGGCCACCGTCACATTC

Supplementary table 1: Sequences of primers used in qRT-PCR experiments.
